# Role of the Endocannabinoid System in the Central Regulation of Nonmammalian Vertebrate Reproduction

**DOI:** 10.1155/2013/941237

**Published:** 2013-09-11

**Authors:** Erika Cottone, Valentina Pomatto, Patrizia Bovolin

**Affiliations:** Department of Life Science and Systems Biology, University of Turin, Via Accademia Albertina 13, 10123 Torino, Italy

## Abstract

The endocannabinoid system (ECS) has a well-documented pivotal role in the control of mammalian reproductive functions, by acting at multiple levels, that is, central (CNS) and local (gonads) levels. Since studies performed in animal models other than mammals might provide further insight into the biology of these signalling molecules, in the present paper we review the comparative data pointing toward the endocannabinoid involvement in the reproductive control of non-mammalian vertebrates, focussing in particular on the central regulation of teleost and amphibian reproduction. The morphofunctional distribution of brain cannabinoid receptors will be discussed in relation to other crucial signalling molecules involved in the control of reproductive functions, such as GnRH, dopamine, aromatase, and pituitary gonadotropins.

## 1. Introduction

The endocannabinoid system (ECS) comprises several components, among which are specific seven transmembrane-domain receptors (i.e., CB1 and CB2 cannabinoid receptors), their exogenous (e.g., Δ^9^-THC) and endogenous ligands (e.g., AEA and 2-AG), and a number of biosynthetic and degradative enzymes (for a review, see [1]). CB1/CB2 receptors are present not only in mammals but also in almost all classes of vertebrates and also in urochordates and cephalochordates, but not in the nonchordate invertebrate phyla. However, enzymes involved in the biosynthesis/inactivation of the endocannabinoids occur throughout the animal kingdom (see the reviews [2, 3]). The fact that the ECS has a rather wide phylogenetic distribution points to a fundamental modulatory role of endocannabinoids in the control of central and peripheral activities, for example, neurotransmission, neural development, hormone release and action, appetite regulation, immunomodulation, cardiovascular and respiratory functions, bone formation, and notably reproduction (for a review, see [4]). Indeed, in both humans and other mammals, the potent negative effects of cannabinoids at peripheral and central levels in embryo implantation, maintenance of pregnancy, and hormonal regulation have been widely documented [5, 6]. In addition, a role for the ECS in the modulation of nonmammalian reproduction has come to light in the last years (for a review, see [7]).

In the present work we will first review the data about the morphofunctional distribution of the cannabinoid receptors in the CNS of nonmammalian vertebrates, namely, teleosts and amphibians. In the second part, we will present comparative data about the involvement of the ECS in the central regulation of reproductive activities of bony fish and amphibians.

## 2. Functional Relevance of Cannabinoid Receptors in Teleost and Amphibian CNS

After the first discovery of CB1 cannabinoid receptors in mammals [8, 9], the cloning of two genes coding for the CB1 receptor subtypes CB1A and CB1B in the bony fish *Fugu rubripes* [10] represented a true milestone in the ECS comparative investigations. Since then, duplicate copies of either the CB1 or CB2 genes (*CNR1, CNR2*) or both have been reported in several teleost species. In the puffer fish *Fugu rubripes*, as already mentioned, two CB1 receptor genes, but only one CB2 gene, are present [10, 11], consistently with the finding of two CB1 genes and one CB2 gene in another puffer fish species, *Tetraodon nigroviridis* [2, 12]. On the other hand, in the zebrafish *Danio rerio*, only one CB1 gene has been detected [13], while two CB2 genes (CB2A and CB2B) are present [14]. In the goldfish *Carassius auratus*, only one CB1 and one CB2 sequences have been cloned so far [15–17]. In the sole *Solea solea*, two CB1 (CB1A and CB1B) were identified [18], while in *Pelvicachromis pulcher* [19], *Sparus aurata* [20], and *Apteronotus leptorhynchus* [21] only one CB1 gene has been cloned. The presence of duplicate genes is a well known phenomenon in teleosts [22] and can be ascribed to a whole-genome duplication that occurred during evolution of the ray-finned lineage before the teleost radiation. At least three different events have been suggested to occur after gene duplication: (1) one gene of the pair evolves as a pseudogene through degenerative mutations and is eliminated because of its dispensability (most frequent event); (2) duplicate genes are preserved and remain functional; in this case, one copy can acquire a mutation conferring a new and beneficial function (neofunctionalization), whereas the other retains the original function; (3) duplicate genes can be preserved and share the multiple functions of the ancestral gene by complementary loss of subfunctions. Thus far, the functional significance of the differential retention of duplicate CB1 or CB2 genes in different teleost lineages is mostly unknown. In one report [14] the expression of CB2A and CB2B has been compared by RT-PCR in various zebrafish tissues, showing a similar distribution with few exceptions (brain and muscle, where CB2A was very low); however, no functional conclusions can be drawn from this study. Recently Palermo et al. [18] showed that in *Solea solea* in a stress paradigm CB1B mRNA levels were significantly reduced in ovary and brain of stressed fish, while CB1A mRNA remained unchanged, suggesting a possible subfunctionalization of the two duplicate genes.

For what concerns amphibians, CB1 cannabinoid receptors have been cloned and characterized in the rough-skinned newt *Taricha granulosa* [23], in the African clawed frog *Xenopus laevis* [24] and in the green frog *Rana esculenta* [25], while the presence of a CB2 gene has been revealed in *Xenopus tropicalis* [2].

Since the distribution of a receptor could give information on its physiological role, a number of studies have been conducted to characterize the localization of cannabinoid receptors.

### 2.1. Distribution of CB1/CB2 Cannabinoid Receptors in Teleost CNS

Although the puffer fish was the first nonmammalian species where CB1 receptors were identified, little is known about cannabinoid receptor distribution in this species. The few available data [10] outline, however, that CB1 receptors are highly expressed in the brain and they are present at lower levels in nonneural tissues, thus indicating an evolutionarily conserved feature common to all vertebrates.

The first data on CB1 receptor distribution in the brain of a teleost were obtained in the African cichlid *Pelvicachromis pulcher* [16, 19]. By using immunohistochemical techniques, abundant CB1-immunostainings were observed throughout the telencephalon, in the preoptic area, lateral infundibular lobes of the posterior hypothalamus and pituitary gland, thus suggesting that cannabinoids affect neuroendocrine mechanisms and might indeed be involved in the control of reproduction. An intense CB1-immunoreactivity was also detected in the pretectum and nucleus glomerulosus of the posterior tuberculum, both transitional areas between pro- and mesencephalon, that are involved in the integration of visual-motor activities in order to orient fish toward preys and elicit appetite [26–28]. In the dorsal mesencephalic tegmentum, some large intensely CB1-immunopositive nerve cells, possibly motor neurons of the III cranial nerve, were observed. In the cerebellum, few granule cells and Purkinje cells were stained, as well as a number of *α*-motoneurons in the spinal cord.

A study in the goldfish [15] showed that CB1-immunoreactivities are distributed through all the forebrain, including the olfactory bulbs. Abundant immunostainings were observed in the inferior lobes of the posterior hypothalamus surrounding the third ventricle lateral recesses; these brain areas are involved in bony fish feeding response, thus providing morphological evidence for the reported involvement of the endocannabinoid system in the goldfish appetite control. The same CB1-immunopositive brain regions are also pivotal for the control of teleost reproduction, the involvement of the ECS being also supported by the finding of CB1-immunopositive fibres in the goldfish infundibulum, but not in the pituitary (Cottone, unpublished data), contrary to what was observed in *Pelvicachromis pulcher*, where pituitary CB1-immunopositive cells were instead observed [29]. In the goldfish, a number of CB1 immunostained cell bodies were also detected in the telencephalon immediately below the ventricular ependyma and were identified as radial glial cells [29, 30]; these cells are very abundant in adult fish, probably due to the absence of astrocytes and ependymal cells and do serve mostly as neural progenitors and newborn neuron migrating scaffolds, as well as neurosteroid-producing cells. The expression of cannabinoid receptors in goldfish radial glial cells does therefore suggest an ECS regulation of neurogenesis in fish, according to what occurred in mammals (for a review [31]).

In zebrafish, CB1 distribution was analysed both in larvae and adult brains, by means of *in situ* hybridization (ISH) [13]. An early CB1 mRNA expression was detected in the preoptic area of the hypothalamus and, later, within the dorsal telencephalon and mesodiencephalon. In postembryonic larvae and adult brain CB1 mRNA is primarily expressed in the dorsal telencephalon, synencephalon, torus longitudinalis, and periventricular hypothalamus, thus suggesting the involvement of the ECS in cognitive processes and neurogenesis.

Very recently, Harvey-Girard et al. [21] presented data on CB1 receptor distribution in the brain of the weakly electric fish *Apteronotus leptorhynchus*. Accordingly with what was observed in the other teleosts, CB1 mRNA is expressed mainly in the telencephalon (especially in subpallial neurons and dorsocentral telencephalon) and in fewer cells in the rest of the brain. CB1 distribution seems to reflect a possible role in the regulation of the electrosensory system and electrocommunication that are particularly developed and important in gymnotiform fish.

Since CB2 receptor genes have been identified in nonmammalian vertebrates more recently than CB1, data about their distribution are scarce. For instance, Rodriguez-Martin et al. [14] have shown in zebrafish by ISH that CB2 mRNA is expressed in the rostral and proximal pars distalis of the pituitary gland, while expression in the brain was only detected by RT-PCR. In goldfish CB2 mRNA has been detected in the brain, although at levels much lower than CB1 mRNA [17] and the immunostaining of goldfish forebrain sections did actually reveal the presence of CB2-immunopositive cells that were identified as radial glial cells, that is, adult neural progenitors [30].

### 2.2. Distribution of CB1/CB2 Cannabinoid Receptors in Amphibian CNS

The first report on the occurrence of the ECS in the amphibian CNS concerned the pharmacological and molecular characterization of CB1 receptors in the urodele amphibian *Taricha granulosa* [23]. In this same species, ISH experiments showed CB1 mRNA expression in the telencephalon, in particular in the olfactory bulb, pallium, bed nucleus of the stria terminalis and nucleus amygdalae dorsolateralis; CB1 mRNA is also expressed in regions of the preoptic area, thalamus, midbrain tegmentum and tectum, cerebellum, and the stratum griseum of the hindbrain [32].

The distribution of CB1 receptor has been also investigated in the CNS of adults and embryos/larvae of the anuran amphibian *Xenopus laevis*. In particular, in whole embryos of *Xenopus laevis* CB1 mRNA was first detected at stage 28, and from stage 41 it appears in the rhombencephalon and thereafter also in the olfactory placodes and then in the olfactory bulbs [33]. In adult *Xenopus laevis*, CB1-immunostainings and CB1 mRNA-positive cells were detected in the olfactory bulb, dorsal and medial pallium, striatum and amygdala, thalamus and hypothalamus, mesencephalic tegmentum, cerebellum and spinal cord [24, 34, 35]. Moreover, CB1 receptors were detected in lactotrophs, gonadotrophs, and thyrotrophs of the pituitary gland [36].

In the green frog *Rana esculenta* CB1 immunostained neurons and nerve fibres are abundant in the telencephalic hemispheres, preoptic area, hypothalamus, and a number of hindbrain areas [37, 38]. Interestingly, fluctuations during the annual sexual cycle of CB1 mRNA expression in various regions of the brain, as well as at the testicular level, have been reported and a possible involvement of the ECS in the regulation of gonadal activity has been postulated [38, 39].

On the basis of their neuroanatomical distribution and relationships with a number of other signalling molecules [34, 35], the amphibian CB1 cannabinoid receptors have been mainly considered modulators in sensory and sensorimotor integrations and endocrine and behavioral outputs. Moreover, since the intense CB1 mRNA ISH staining found in *Taricha granulosa* amigdaloid complex [32] well matched the strong CB1-immunoreactivity and mRNA expression in the corresponding nucleus of *Xenopus laevis* [24, 34], the endocannabinoid-mediated modulation of fear, anxiety, and stress responses has also been postulated.

## 3. The Endocannabinoid System Is Involved in the Central Regulation of Teleost and Amphibian Reproductive Functions

**Figure 1:**
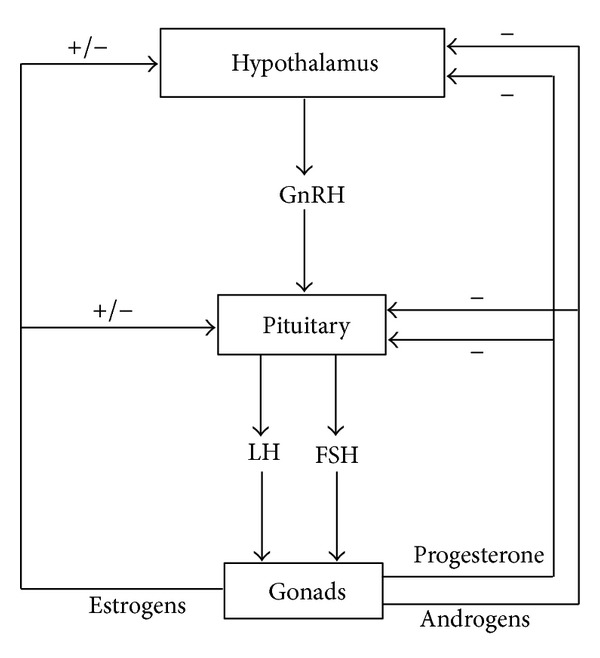
Schematic representation of the hypothalamus-pituitary-gonadal (HPG) axis.

The reproductive functions are regulated by the neuroendocrine system, so that a tight crosstalk between the hypothalamus, pituitary, and gonads takes place (Figure 1). In particular, the decapeptide GnRH is released from the hypothalamus and stimulates pituitary to release gonadotropins. FSH and LH act then at the gonadal levels, regulating spermatogenesis, oocyte growth, and steroidogenesis.

Cannabinoids exert potent negative effects upon both experimental animal and human reproduction, affecting gonadotropin synthesis and release, gonadic steroid production, spermatogenesis, ovulation, embryo development and implantation, sexual behaviour [6, 40, 41]. In vertebrates ECS modulates reproductive functions by acting at multiple levels (for a review, see [42]). Centrally, ECS negatively affects the secretion of pituitary gonadotropin hormones, by acting at the hypothalamic level. Also, a direct action on pituitary is possible, since AEA regulates *in vitro* pituitary hormone secretions in rat [43], and CB1 receptor has been found in mammalian pituitary anterior lobe [44] and in *Xenopus laevis* PRL and FSH cells [36]. The fact that CB1 receptors were detected in the Leydig cells of mouse testis and the endogenous cannabinoid anandamide (AEA) suppressed testosterone secretion by testes in normal but not in CB1 knockout mice [43] was the first evidence that cannabinoids do modulate reproductive functions also at the peripheral level. Recent data also point out a role for CB2 receptor, whose presence was observed in rat and human ovaries [45, 46], in human oocytes [47] and mouse spermatogonia [48]. In addition, endocannabinoids control sperm motility and/or acrosomic reaction [49–53], as well as ovulation, implantation, embryonic development, foetal growth, lactation (see the reviews [6, 54]). It is noteworthy that also in nonmammalian vertebrates basic components of the endocannabinoid system were found and characterized both in the brain and in the gonads. CB1 mRNA expression was in fact detected in the gonads of *Carassius auratus* and *Pelvicachromis pulcher* [55] and *Sparus aurata* [20] and *Danio rerio* [56], as well as* Xenopus laevis* [55]. In *Rana esculenta* the occurrence of both CB1 receptors and FAAH in the gonads was reported and CB1 levels have been shown to fluctuate during the annual sexual cycle [53], consistently with the observations in the brain [38, 39]. Also, the ECS inhibits male courtship clasping behaviour in the newt *Taricha granulosa* [23] and interferes with mammalian copulatory behaviour [6]. At the moment there are scarce or no available data regarding CB2 receptor in nonmammalian vertebrate gonads, with the exception of the teleost goldfish, where CB2 mRNA was found in testis and ovary [17].

**Figure 2:**
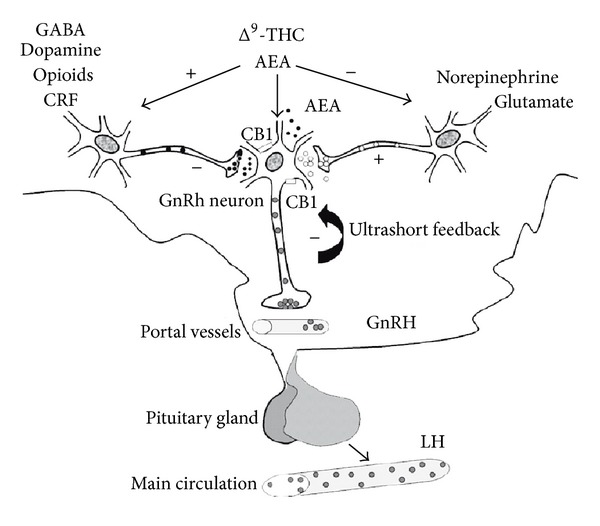
Schematic representation of direct and indirect effects of the ECS in the control of GnRH release (from [57]).

At the central level, the action of the ECS on the hypothalamus has been first demonstrated by the observation that both exogenous and endogenous cannabinoids are responsible for the decrease of circulating LH and sexual steroids, by acting through a CB1-mediated inhibition of GnRH-I (formerly called *mammalian*GnRH) discharge (for a review, see [57]). The cannabinoid ligands might inhibit GnRH secreting neurons by activating specific signalling circuitries (e.g., GABA, dopamine, CRF, opioid) and inhibiting others (e.g., glutamate, norepinephrine). Moreover, since immortalized hypothalamic GnRH neurons are capable of releasing endogenous cannabinoids such as AEA and 2-AG and do possess CB1 and CB2 receptors [58], a direct neural control of GnRH-I release has been postulated (see Figure 2). Since GnRH is a key molecule in the gonadotropic regulation of all vertebrates, studies on the morphofunctional relationships between ECS and GnRH were conducted also in nonmammalian vertebrates. In particular, in *Pelvicachromis pulcher*, *Carassius auratus*, *Solea solea*, and *Danio rerio*, a close contiguity between CB1-immunostainings and GnRH-III- (previously called *salmon*GnRH) immununoreactive cell bodies and nerve fibers was observed in brain areas that are pivotal for the control of reproduction, such as the basal telencephalon, the preoptic area, and the hypothalamus ([16, 55, 59] and Cottone, unpublished data). Consistently, in *Rana esculenta* and *Xenopus laevis*, the codistribution of CB1- and GnRH-I-immunoreactivity occurred in brain areas corresponding to those described in the above teleost species [37, 55]. In particular, a subset of frog GnRH-I-immunoreactive neurons in the septum and preoptic area, together with nerve fibres and terminals in the median eminence of the neurohypophysis, were found CB1-immunopositive [38]. Interestingly, during the frog annual sexual cycle, GnRH-I mRNA and CB1 levels have opposite expression profiles in the telencephalon and diencephalon [38, 60]. Also, anandamide is able to inhibit GnRH-I and GnRH-II synthesis and to affect GnRH receptors expression in the diencephalon, as well as in the testis; at the same time, a GnRH agonist inhibits the synthesis of GnRH-I mRNA and induces an increase in CB1 transcription [38, 61, 62], thus suggesting reciprocal relationships between the ECS and the GnRH system.

In line with dopaminergic control of fish reproduction through inhibition of both PRL release and gonadotropin (GTH-I and GTH-II) synthesis [63], CB1-immunoreactivities were found codistributed with TH- (the dopamine biosynthetic rate-limiting enzyme) immunopositive neurons and fibres in the basal telencephalon/preoptic area of *Pelvicachromis pulcher* and *Carassius auratus* [16]. Also, in the diencephalic paraventricular organ (PVO) of the goldfish a number of CB1-immunopositive cerebrospinal fluid (CSF) contacting neurons were found closely adjacent to the TH-positive neurons that innervate the neurointermediate and the distal lobes of the teleost pituitary, thus indicating a CB1-mediated control of PVO dopaminergic neurons and, consequently, a direct or indirect regulation of pituitary activity [29].

**Figure 3:**
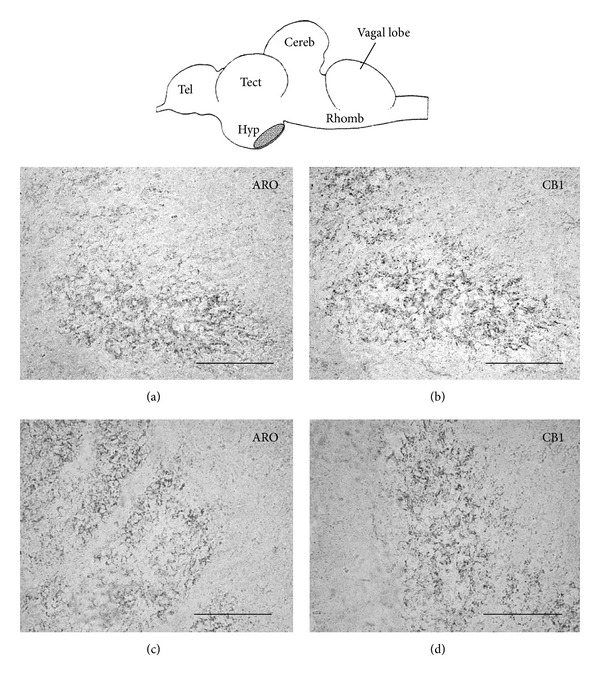
Aromatase/CB1 immunoreactivities in the goldfish lateral recesses of the hypothalamus (depicted in the schematic draw of the goldfish brain, lateral view). (a), (b) and (c), (d): consecutive coronal sections showing in (a) and (c) a large number of aromatase-immunopositive radial glial cell processes codistributed with ((b) and (d), resp.) abundant CB1-immunopositive fibers and cell processes. Calibration bars: 100 *μ*m. Cereb: cerebellum; hyp: hypothalamus; rhomb: rhombencephalon; tect: optic tectum of the mesencephalon; tel: telencephalon; vagal lobe: lobe of the X cranial nerve.

Another molecule deeply involved in the reproductive physiology is aromatase (estrogen synthase), the enzyme that catalyzes the transformation of androgens into estrogens (for a review, see [64]). Aromatase is expressed both in gonads and brain; brain aromatase is essential for testosterone-mediated regulation of physiological and behavioural processes, such as sexual differentiation of the brain, activation of male sexual behaviour, and regulation of gonadotropic hormone secretion, as well as neurogenesis. Teleost fish do possess two aromatase genes, codifying for aromatase A, that is specifically expressed in the gonads, and aromatase B, that is strongly expressed in the brain. Indeed, teleosts, compared to other vertebrates, show remarkably high levels of brain aromatase activity and protein and gene expression, due to an autoregulatory loop through which estrogens and aromatizable androgens upregulate aromatase expression [65]. In zebrafish abundant aromatase-positive cells have been observed in the olfactory bulbs, telencephalon, preoptic area, and the hypothalamus, as well as in the thalamus, optic tectum, and around the fourth ventricle [66–68]. Interestingly, brain aromatase expression and activity fluctuate seasonally and with the reproductive state. Peculiarly, in teleosts brain aromatase is not expressed by neurons, as seen in mammals and birds. Instead, it is specifically expressed by radial glial cells [66], the cells that sustain the high neurogenic activity of adult fish; thus, in fish, aromatase is likely to be the key molecule enabling brain growth and brain sexualization throughout life. Given the role of aromatase in reproductive physiology, we evaluated the possible existence of a crosstalk between the ECS and brain aromatase, by analyzing the codistribution of CB1 cannabinoid receptors and aromatase in brain sections of both zebrafish and goldfish. In the preoptic area and periventricular grey of the hypothalamic inferior lobes, a tight contiguity between the two markers was indeed found (Figure 3 and data not shown), thus suggesting a CB1-mediated regulation of aromatase activity at least in bony fish.

## 4. Concluding Remarks

The data reported in teleosts and amphibians strongly support the modulatory role of brain ECS on several neural circuits, including those involved in the control of reproductive functions.

Although the general physiological role of endocannabinoids is far from being understood in nonmammalian vertebrates, the investigations in organisms different from mammals might provide new insights into the cannabinoid biology. Basic information on the ECS derived from comparative investigations in invertebrates and/or anamniote vertebrates, besides bearing value for evolutionary and wildlife biological studies, could contribute to a better understanding of the mechanisms of action of cannabinoid-related molecules and stimulate the development of new strategies for their therapeutic use in humans.
